# Experimental Investigation of Seismic Performance of Precast Concrete Wall–Beam–Slab Joints with Overlapping U-Bar Loop Connections

**DOI:** 10.3390/ma16093318

**Published:** 2023-04-23

**Authors:** Feng Chen, Zhiwu Yu, Yalin Yu, Zhipeng Zhai, Qun Liu, Xiao Li

**Affiliations:** 1 School of Civil Engineering, National Engineering Research Center of High-Speed Railway Construction Technology, Engineering Technology Research Center for Prefabricated Construction Industrialization of Hunan Province, Central South University, 68 South Shaoshan Road, Tianxin District, Changsha 410075, China; 2 Department of Civil Engineering, University of Nottingham, Nottingham NG7 2RD, UK; 3 Earthquake Engineering Research & Test Center (EERTC), Guangzhou University, 230 West Waihuan Road, Panyu District, Guangzhou 510405, China; 4 Qingdao Luneng Real Estate Co., Ltd., 265 Haikou Road, Laoshan District, Qingdao 266061, China

**Keywords:** precast structures, overlapping U-bar loop connections, wall–beam–slab joints, seismic performance, quasistatic test

## Abstract

In the era of energy conservation and environmental protection, as well as the industrialization of buildings, precast concrete (PC) structures have been developed and increasingly applied in construction industries due to their advantages of outstanding workability and ecofriendliness. In order to verify the reliability of overlapping U-bar loop connections and a modified form of these connections, and study the seismic performance of PC wall–beam–slab joints with these connection methods, three full-scale wall–beam–slab joints were designed and tested under low reversed cyclic loading, including one cast-in-place (CIP) specimen and two PC specimens. Based on the test results, the seismic performance of the PC joints was studied by comparing their damage process, hysteretic loops and skeleton curves, load-carrying capacity, ductility, equivalent stiffness, and energy dissipation with those of the CIP joint. After analyzing the experimental results, the following conclusions can be drawn: the overlapping U-bar loop connection and its modified form are effective and reasonable; the specimen with the modified connection form showed slightly better mechanical properties; the failure mode of the PC joints was consistent with that of the CIP joint; and the generation, distribution, and development of cracks in the PC specimens were similar to those in the CIP specimen. In addition, the stiffness of the PC joints was similar to that of the CIP joint, and the load-carrying capacity, ductility, and energy dissipation of the PC joints were better than those of the CIP joint. Moreover, the research in this paper can also provide some guidance for assembling wall–beam–slab joints in PC shear wall structures.

## 1. Introduction

### 1.1. Background and Previous Investigations

The development of precast buildings is a significant change in construction methods which is conducive to saving resources and energy, reducing construction pollution, and deeply promoting the process of construction industrialization [1,2]. Thus, the application and mechanical properties of PC structures have attracted great attention, and a large number of studies have been conducted on these structures’ seismic performance [3,4,5,6,7]. As an important type of PC structure, the precast shear wall structure has received great attention and has been studied extensively and widely used in many public and civil buildings. Moreover, various precast shear wall structures with different steel bar connection methods and joint construction measures have also been proposed by researchers. At present, grouted splice sleeve connections [8,9], pore-forming grouted lap-spliced connections [10,11], and unbonded post-tensioned connections [12,13] have been widely applied in many different practical construction projects as very common steel bar connection methods. The advantages of each of the connection methods enable them to be effectively used in PC structures, but there are also some disadvantages which adversely affect the mechanical properties of PC structures, or increase the cost, difficulty, and complexity of construction [11,12,13,14,15,16,17,18]. Therefore, after evaluating the advantages and disadvantages of the existing connection methods, and based on a series of studies, we propose a new connection method—the overlapping U-bar loop connection, which provides an alternative effective method for the assembly of different components of PC shear wall structures. This method avoids many complicated construction operations such as the grouting of sleeves and the tensioning of prestressing tendons on the basis of ensuring that the structures’ mechanical properties, such as bearing capacity, stiffness, ductility, and energy dissipation performance are close to those of CIP structures. Compared with other existing methods, this not only greatly simplifies the construction process, but also makes quality inspection simpler and more effective. Furthermore, the cost of this method is quite low [14].

**Figure 1:**
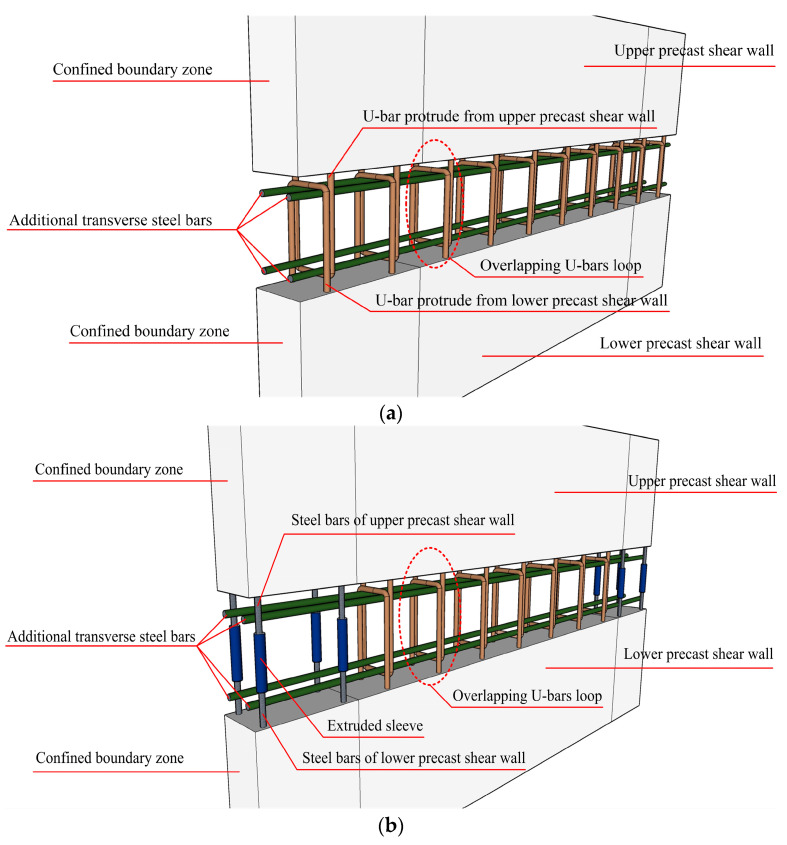
Schematic diagram of overlapping U-bar loop connections. (**a**) Pure overlapping U-bar loop connections. (**b**) Overlapping U-bar loop connections combined with extruded sleeve connections.

Nonetheless, there are still some aspects that need to be improved [19,20,21]. Therefore, in order to further optimize the overlapping U-bar loop connection in PC shear walls, make up for its shortcomings in terms of mechanical properties, and take full advantage of its seismic capacity, modified form-overlapping U-bar loop connections combined with extruded sleeve connections are proposed by referring to previous studies on PC columns [22]. The specifics of these two connection forms are shown in Figure 1. Vertical distributed steel bars extend out of the precast shear walls to form U-bar loops. When assembling, the U-bars of the upper precast wall and the lower precast wall are lap-spliced to each other to form rectangular closed loops. After that, four additional transverse steel bars are inserted at the corners of the closed loops to strengthen the overlapping U-bar loop connection. The difference between the modified form and the original pure overlapping U-bar loop connection form lies in the connection method applied to the longitudinal steel bars located in the confined boundary zone of the shear walls. For the pure overlapping U-bar loop connection form, longitudinal steel bars located in the confined boundary zone are connected by overlapping U-bar loops, while for overlapping U-bar loop connections combined with the extruded sleeve connection form, longitudinal steel bars in the confined boundary zone are connected by extruded sleeves. The overlapping U-bar loop connection mainly relies on the bonding action between the vertical section of the overlapping U-bars and the concrete and the confinement action of the horizontal section of the U-bars and the additional transverse steel bars on the intermediate concrete to transfer the load. In general, the overlapping U-bars, four additional transverse steel bars, and the concrete surrounded by these steel bars form a concealed beam, which mainly plays the role of transferring the load and bearing the shear force of the component. Moreover, in order to increase the friction between the PC concrete and post-cast concrete, the surface of the precast component in contact with the post-cast concrete is roughened, which makes it more capable of resisting the shear force.

### 1.2. Motivation

At present, most existing studies mainly focus on the vertical and horizontal connection of PC shear walls, whereas little attention is paid to the seismic performance of wall–beam–slab joints [23,24]. This paper introduces an experimental program on PC shear wall–beam–slab joints characterized by overlapping U-bar loop connections. In order to further verify the connection performance of the pure overlapping U-bar loop connection and the modified form-overlapping U-bar loop connections combined with extruded sleeve connections, and study the seismic performance of the PC joints with these connection forms, three full-scale joints were designed, including one CIP joint, one PC joint with pure overlapping U-bar loop connections, and one PC joint with the modified form. A quasistatic test was conducted to investigate the mechanical behavior of the PC and CIP specimens under low reversed cyclic loading. Based on the test results, some important indicators such as the load-carrying capacity, deformation, ductility, stiffness, and energy dissipation performance of the PC joints were compared with those of the CIP joint to verify the rationality of the application of overlapping U-bar loop connections in PC shear wall–beam–slab joints. In this way, the seismic performance of these two PC joints with different connection forms was also studied.

**Figure 2:**
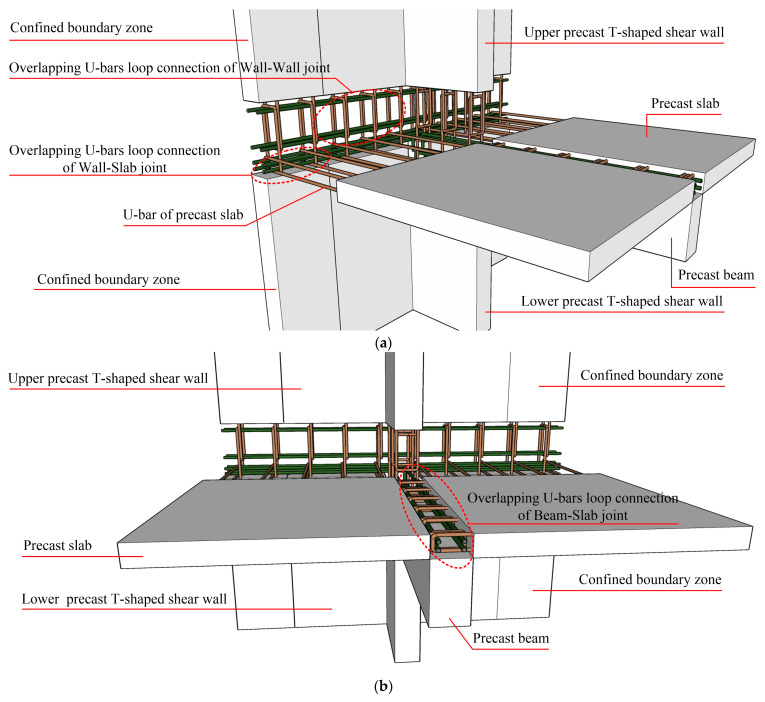
Schematic diagram of overlapping U-bar loop connections of wall–beam–slab joint. (**a**) Front view. (**b**) Lateral view.

The specifics of PC shear wall–beam–slab joints with overlapping U-bar loop connections are shown in Figure 2. The details of the overlapping U-bar loop connections between the slab and the shear wall as well as the slab and the beam are similar to the details mentioned in Section 1.1. Considering that if the longitudinal steel bars extending out of the PC beam are U-shaped, this may cause the position to conflict with the steel bars extending out of the upper and lower PC shear walls, in order to ensure the feasibility and convenience of construction, the longitudinal steel bars extending out of the PC beam are directly anchored into the PC shear wall with adequate development length.

## 2. Experimental Program

### 2.1. The Specimens

**Figure 3:**
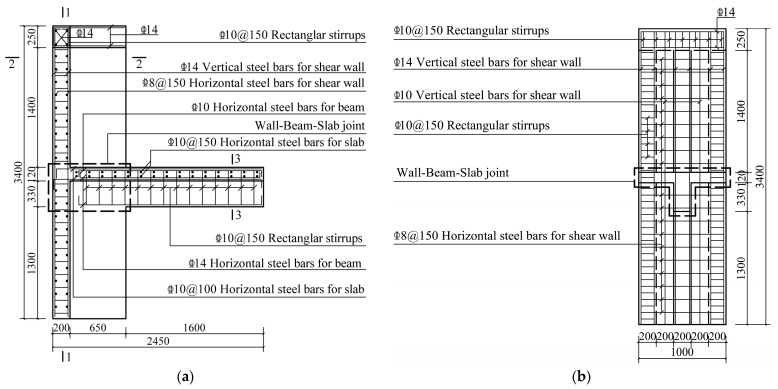

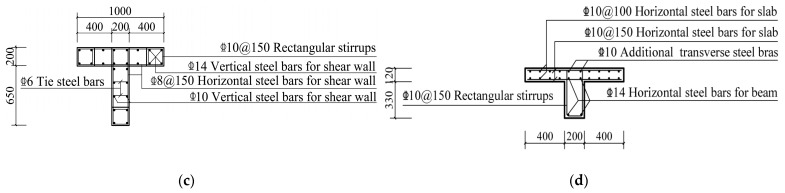
Dimensions and steel bar layout of JD1. (**a**) Front view; (**b**) 1-1 profile; (**c**) 2-2 profile; (**d**) 3-3 profile.

**Figure 4:**
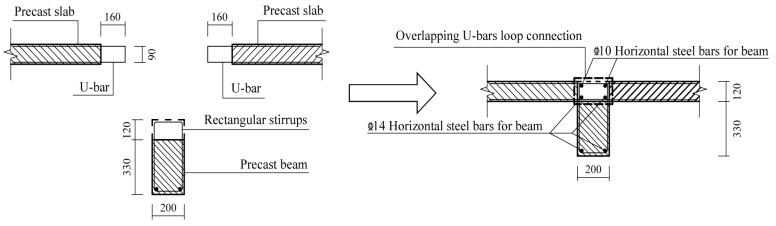
Assembly construction schematic diagram of beam–slab joint.

**Figure 5:**
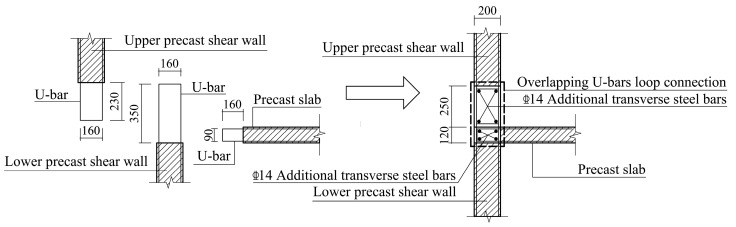
Assembly construction schematic diagram of wall–wall–slab joint.

**Figure 6:**
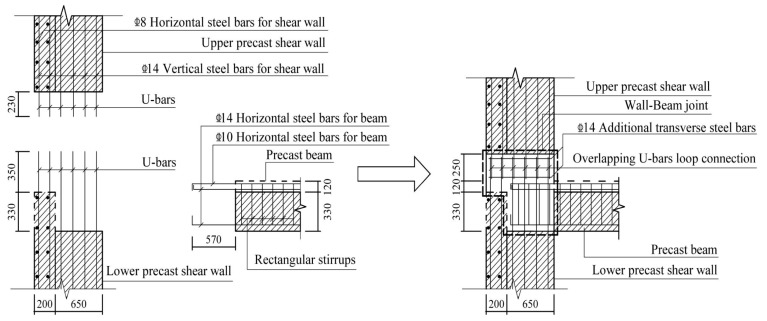
Assembly construction schematic diagram of wall–beam joint.

**Figure 7:**
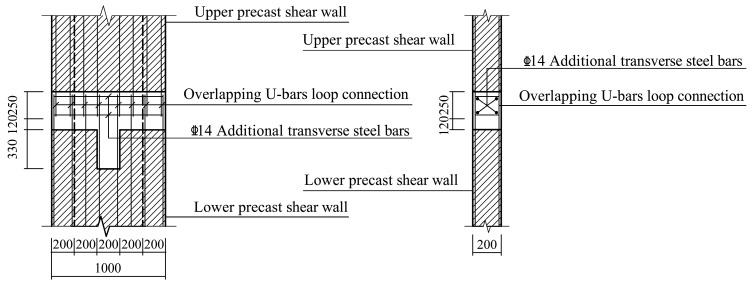
Schematic diagram of overlapping U-bar loop connection of JD2.

**Figure 8:**
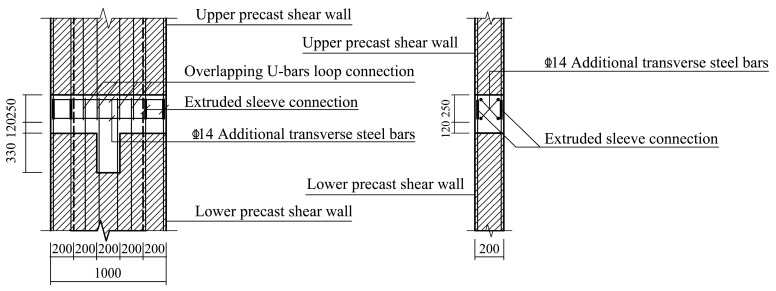
Schematic diagram of overlapping U-bar loop connections combined with extruded sleeve connections of JD3.

Three full-scale specimens were designed for the quasistatic test. The specimen model was derived from an edge joint of the middle floor of a practical project. Specifically, the monolithic CIP joint designed as the comparison specimen was named JD1; the two precast specimens were named JD2 and JD3. Except for the longitudinal steel bars extending out of the beam, which were directly anchored into the web of the T-shaped shear wall, all the other precast components of JD2 were connected through overlapping U-bar loops. For JD3, the modified connection form was applied. Except for the vertical steel bars in the confined boundary zone of the upper and lower shear walls, which were connected by extruded sleeves, connections of other parts were the same as those of JD2. The connection methods applied between different precast components are shown in Table 1. Except for the difference in connection methods, the steel bar layouts and the geometrical dimensions of all three specimens were identical. The axial loads were transferred through the RC hidden beam at the top of each shear wall with dimensions of 200 mm in width and 280 mm in height. Figure 3 shows the detailed dimensions and steel bar layout of CIP specimen, and Figure 4, Figure 5, Figure 6, Figure 7 and Figure 8 briefly present the connection methods and assembly construction procedures of PC specimens.

### 2.2. Material Properties

Relevant mechanical parameters of concrete and steel bars with different diameters are listed in Table 2 and Table 3, respectively. HRB400 was used for steel bars, and the CIP specimen was fabricated with ordinary concrete. As for PC specimens, the nominal concrete compressive strength of all precast components was the same as that of the CIP specimen, which was 30 MPa, while for post-cast concrete in the assembly region, this value was 40 MPa. However, the actual compressive strength of post-cast concrete was decreased due to the addition of an expansion agent in the production process.

### 2.3. Test Setup and Loading Method

#### 2.3.1. Test Setup

**Figure 9:**
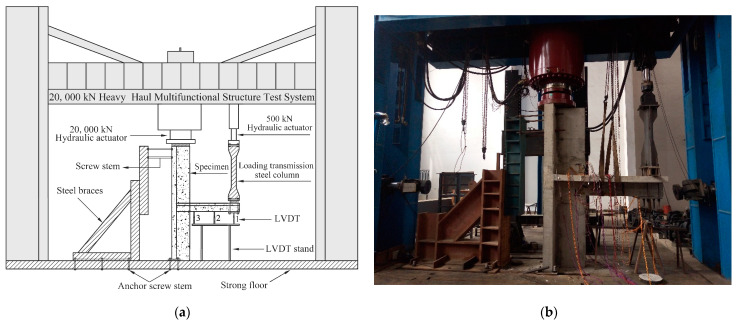
Test setup. (**a**) Schematic. (**b**) Photo.

The experiment was carried out at the line-bridge-tunnel static laboratory of the National Engineering Laboratory for High Speed Railway Construction Technology. Details of the test setup are presented in Figure 9. The top of the specimens was connected with the steel brace by threaded bars, whilst the bottom of the steel brace was fixed on the laboratory floor by anchored threaded bars. In addition, the bottom of the shear wall was fixed on the laboratory floor by four anchored screw stems. In this way, the horizontal movement of specimens was limited. When loading, the axial load was imposed on the steel plate placed on the top of the shear wall by the 20,000 kN hydraulic actuator, consequently transmitting the load to the specimen evenly. Meanwhile, the low reversed cyclic load was applied at the end of the beam by a 500 kN hydraulic actuator. On account of the distance from the end of the beam to the 500 kN hydraulic actuator being greater than the maximum stroke of the actuator, a loading transmission steel column was connected to the actuator, whilst the loading transmission steel column was fixed at the end of the beam by screws so that the low reversed cyclic load imposed by the actuator could be transmitted to the specimens effectively.

#### 2.3.2. Loading Method

According to the code “Technical specification for concrete structures of tall building” [25] and “Code for Design of Concrete Structures” [26], the axial compression ratio is expressed as Equation (1).

(1) $$n=1.2\times \frac{N}{f_{c}\times A_{w}}$$

where *n* is the design value of the axial compression ratio. According to the codes mentioned above, 0.4 was selected as the axial compression ratio of the test. *f_c_* is the design value of the compressive strength of concrete, *A_w_* is the gross cross-section area of specimens, and *N* is the standard value of axial force. According to the “Load code for the design of building structures.” [27], the design value of axial force is equal to the standard value multiplied by the factor 1.2. Based on Equation (1)*,* the standard axial force imposed on the top of the shear wall was calculated to be 1555 kN and remained constant during the test.

**Figure 10:**
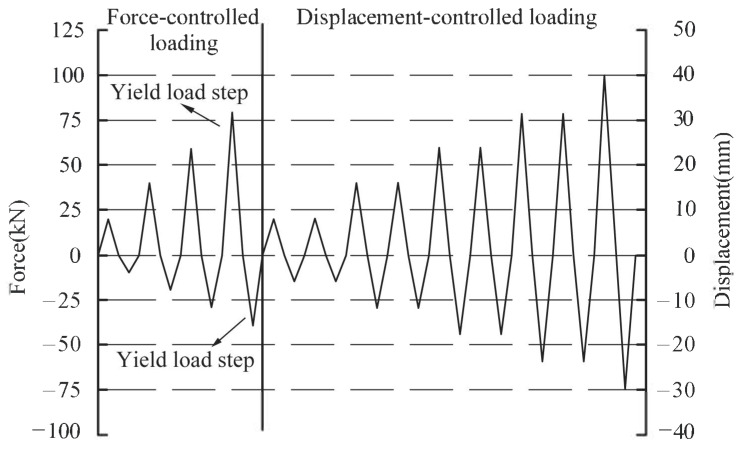
Loading process.

Meanwhile, a low reversed cyclic load was imposed on the end of the beam using force–displacement mixed control loading system. The loading protocol is indicated in Figure 10. Before specimens yielded, the force-controlled loading mode was adopted. It was determined that the vertical downward load was positive and the vertical upward load was negative. Since the beam–slab was equivalent to a T-shaped beam in the process of loading, the load-carrying capacity of the beam–slab under upward loading was significantly asymmetric compared with that under downward loading. Therefore, the downward test load was designed to be approximately twice as much as the upward test load after trial calculation. After specimens yielded, the loading mode was switched to displacement-controlled loading. The maximum positive and negative displacements of the 500 kN hydraulic actuator when specimens yielded were taken as the initial and basic loading displacements Δ*_a_*, respectively. In addition, the loading displacement of each step in the displacement-controlled stage was increased by Δ*_a_*. When the vertical resistance of specimens dropped to 85% of the peak load or specimens showed obvious serious damage, the loading was stopped.

#### 2.3.3. Measurement Items

The main measurement items included loads of each loading step, vertical displacements of the beam, and strains of steel bars in the vicinity of the joint region. As shown in Figure 9, three linear variable displacement transducers (LVDTs) were mounted on the LVDT stand to measure the vertical displacement of different parts of the beam. LVDT 1 was located under the loading position to measure the vertical displacement of the beam end, which was used to draw hysteretic loops and skeleton curves of specimens. During the whole test process, the displacement measured by the LVDT was consistently taken as the actual displacement of beams for data analysis and experiment discussion, while the actuator displacement was only used as the basic loading displacement for staged loading after specimens yielded.

**Figure 11:**
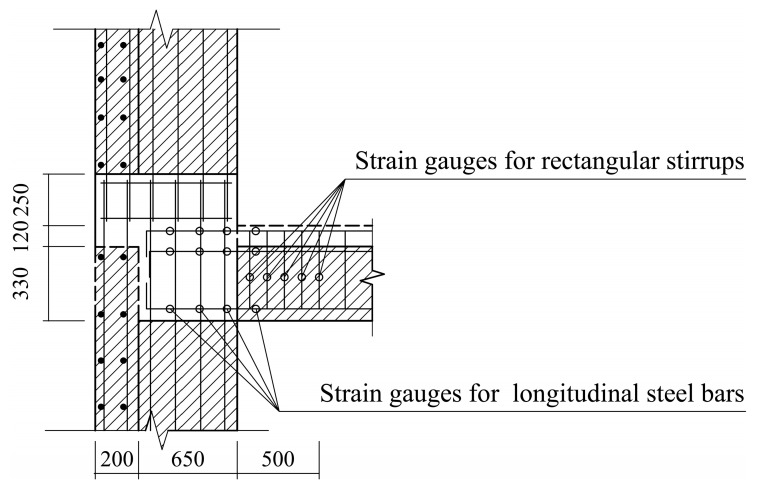
Schematic diagram of strain gauge layout.

As depicted in Figure 11, in the joint region where the beam is connected to the web of the T-shaped shear wall, dozens of strain gauges were attached at 200 mm intervals on the longitudinal steel bars of the beam to monitor the variation of the steel bar strains at different positions with the increase in load. 

## 3. Experiment Results and Discussion

Based on the requirements of the “Specification for Seismic Test of Buildings” [28], the definitions of the relevant mechanical characteristics can be illustrated as follows. The load applied and the deformation generated when cracks initially occurred were defined as cracking load *F_cr_* and cracking displacement Δ*_cr_*, respectively. Referring to the corresponding parameters of the steel bars shown in Table 3, it was considered that the specimens yielded when the maximum strain of the longitudinal steel bars in the beams reached 2300 με; the corresponding load and deformation were defined as yield load *F_y_* and yield displacement Δ*_y_*, respectively. The maximum load that could be achieved during the test and the corresponding displacement were defined as peak load *F_p_* and peak displacement Δ*_p_*, respectively. After the test reached the peak load, the vertical resistance of the specimens decreased with the increase in displacement and the loading step. According to the general consideration, it was thought that the specimens reached the ultimate state when the vertical resistance of the specimens dropped to 85% of the peak load or the specimens showed obvious serious damage. The load and displacement at this moment were defined as ultimate load *F_u_* and ultimate displacement Δ*_u_*, respectively.

### 3.1. Damage Process and Failure Mechanisms

#### 3.1.1. Observations for the JD1 Specimen

In the force-controlled phase, the steps of downward loading were 20 kN, 40 kN, 60 kN, and 80 kN, while the upward loading value of each step was half of the downward loading value; the load of each step was reciprocated once. When the load reached 40 kN, cracks occurred initially at the top of the slab and were located at the construction joint. When the load reached −30 kN, many vertical cracks occurred at the bottom of the beam and were located in the vicinity of the construction joint. With the load increasing, more parallel cracks were generated at the top of the slab and gradually extended from the junction of the shear wall and slab to both ends. JD1 yielded when the load attained 80 kN. After this loading step, the loading mode was switched to displacement-controlled loading.

**Figure 12:**
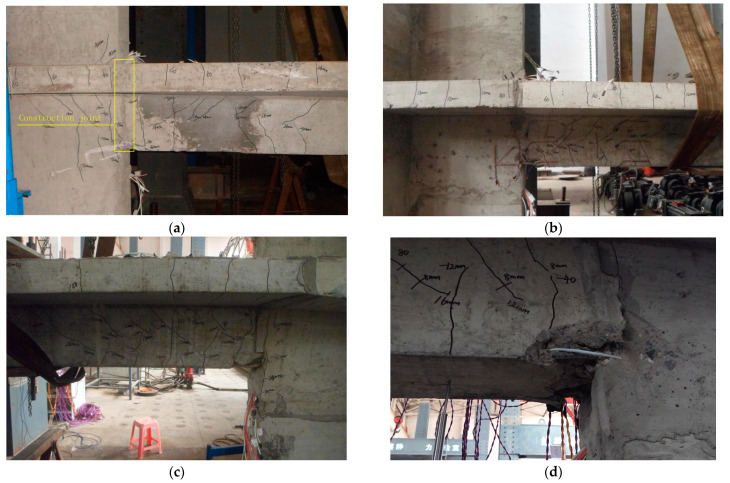

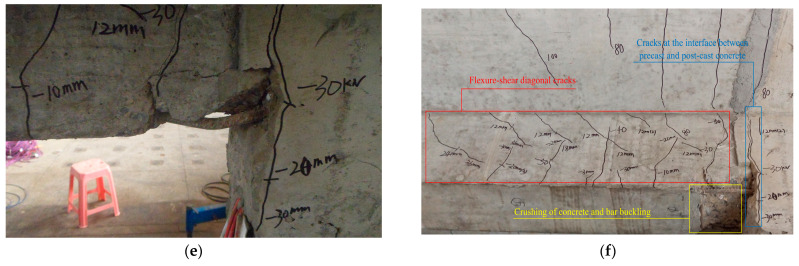
Typical damage of specimens. (**a**) Crack distribution of JD1. (**b**) Crack distribution of JD2. (**c**) Crack distribution of JD3. (**d**) Crushing of concrete. (**e**) Bar buckling. (**f**) Flexure–shear diagonal cracks and cracks at the interface between precast and post-cast concrete.

At the end of the 2Δ*_a_* loading cycles, diagonal cracks of approximately 45 degrees occurred at the beam and shear wall. The distribution range of the cracks continued to expand, accompanied by the original cracks extending sequentially during the 3Δ*_a_* and 4Δ*_a_* loading cycles; the displacement of JD1 reached the peak value of 29.8 mm during the 4Δ*_a_* loading step. In the duration of the 5Δ*_a_* loading cycles, vertical cracks occurred in the T-shaped shear wall when the loading displacement approached −30 mm; several diagonal cracks were generated in the upper shear wall when the loading displacement increased to 40 mm, and the actual ultimate displacement of JD1 was achieved at 30.68 mm, accompanied by the concrete at the bottom of the beam being crushed and the vertical resistance decreasing to 83% of the peak load. The crack distribution of JD1 is shown in Figure 12a.

#### 3.1.2. Observations for the JD2 Specimen

When the load reached 40 kN, initial cracks on JD2 appeared at the top of the slab where the beam–slab was connected to the web of the T-shaped shear wall. After the fourth force-controlled loading cycle was completed, JD2 entered the yielding state. In this stage, the load approached 80 kN, several approximate parallel vertical cracks occurred in the slab, and the crack spacing was about 160 mm. There were a few cracks that occurred in the beam and shear walls. 

During the loading process of the 2Δ*_a_* loading cycles, many vertical cracks appeared at the bottom of the beam when the loading displacement reached −12 mm. After that, in the duration of the 3Δ*_a_* loading cycles, the cracks in the slab extended into the beam and developed diagonally when the loading displacement attained 24 mm, which indicated that the flexure–shear diagonal cracks occurred in the beam under the shear force. When the loading displacement reached 4Δ*_a_*, the load-carrying capacity of the specimen reached its peak value. During this process, there were no new cracks that occurred, whereas the original cracks developed and extended dramatically. By the time the loading displacement reached 5Δ*_a_*, the concrete at the bottom of the beam was crushed and peeled off. Once the loading displacement continually increased to 6Δ*_a_*, the concrete was crushed and peeled off much more seriously, which resulted in the steel bars being exposed. The cracks penetrated through the interface between the precast and post-cast concrete. Meanwhile, the vertical resistance dropped to 79% of the peak load and the actual deformation of JD2 reached the maximum value. The crack distribution of JD2 is shown in Figure 12b.

#### 3.1.3. Observations for the JD3 Specimen

Similar to JD1 and JD2, initial cracks on JD3 occurred when the load reached 40 kN. In the duration of the fourth force-controlled loading cycle, a few steel bars yielded when the load approached 80 kN. According to the load–displacement curve and the strains of the steel bars, it was generally considered that the specimen yielded. 

After the 1Δ*_a_* loading cycles, several diagonal cracks occurred in the beam. During the upward displacement loading phase of the 2Δ*_a_* loading cycles, vertical cracks at the bottom of the beam extended upward and intersected with the diagonal cracks that had appeared earlier. Few new cracks occurred during the subsequent loading process, whereas the original cracks continuously expanded. When the loading displacement reached 4Δ*_a_*, the load-carrying capacity of the specimen attained its peak value. Finally, during the 5Δ*_a_* loading cycle, when the loading displacement of the actuator reached 60 mm, the longitudinal bars bulged and were exposed with the crushing of the concrete at the bottom of the beam, and the vertical resistance decreased to 88% of the peak load. As the specimen had been seriously damaged, the test was terminated. The crack distribution of JD3 is shown in Figure 12c. Moreover, the typical damage of specimens is also shown in Figure 12e,f.

### 3.2. Hysteretic Loops and Skeleton Curves

**Figure 13:**
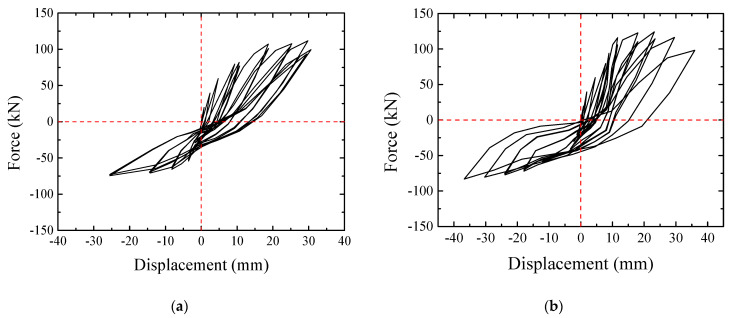

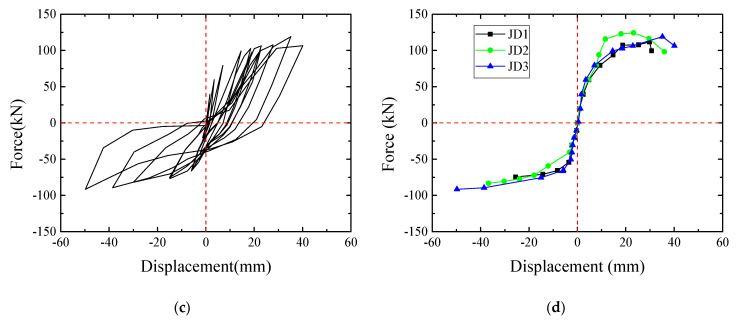
Hysteretic loops and skeleton curves of specimens. (**a**) Specimen JD1. (**b**) Specimen JD2. (**c**) Specimen JD3. (**d**) Skeleton curves of specimens.

The hysteretic loops and skeleton curves are drawn based on the load applied in every loading cycle and the corresponding displacement of the beam edge measured by LVDT 1. As demonstrated in Figure 13a–c, the hysteretic properties of all the specimens were close to each other; the hysteretic loops all presented an “inverse S” shape and exhibited a similar and obvious pinching effect. Besides, the hysteretic loops were much fuller under downward loading than under upward loading. 

The skeleton curves of the three specimens are compared in Figure 13d. It can be seen from the figure that the skeleton curves of the PC specimens and the CIP specimen were extremely similar to each other, while the PC specimens showed slightly better load-carrying capacity. The downward loading phase of the skeleton curves included the ascending, gentle, and descending stages, while the upward loading phase of the skeleton curves only included the ascending and gentle stages. 

### 3.3. Load-Carrying Capacity, Displacement, and Ductility

The resistance and deformation of all the specimens at different loading steps are shown in Table 4. The cracking load and yield load of PC specimens were extremely close to that of the CIP specimen, whereas the cracking displacement and yield displacement were smaller than that of the CIP specimen. The peak load and ultimate load of PC specimens were slightly larger than that of the CIP specimen; likewise, the peak displacement and ultimate displacement of PC specimens were also larger. In order to characterize the ductility of the specimens, the ratio of the ultimate displacement Δ*_u_* to the yield displacement Δ*_y_* was defined as the ductility coefficient Δ*_u_/*Δ*_y_*. Since none of the specimens reached their ultimate upward load and displacement under upward load, the ductility coefficients under downward load were taken as the ductility coefficients of the specimens, which are presented in Table 4. The ductility of JD2 and JD3 was clearly better than that of JD1.

Generally, the weaknesses of PC specimens are caused by the discontinuity of the steel bars and the existence of the precast-to-post-cast concrete interfaces. Nevertheless, the effect of this weakening is limited. Instead, the presence of overlapping U-bar loop connections and additional steel bars not only made the steel bar ratio increase but also had a strong confinement effect on the post-cast concrete. Meanwhile, the strength of the post-cast concrete was also increased. The combined effect of these measures compensated for the weaknesses, leading to a better performance in terms of the load-carrying capacity and ductility of the PC joints.

### 3.4. Equivalent Stiffness

**Figure 14:**
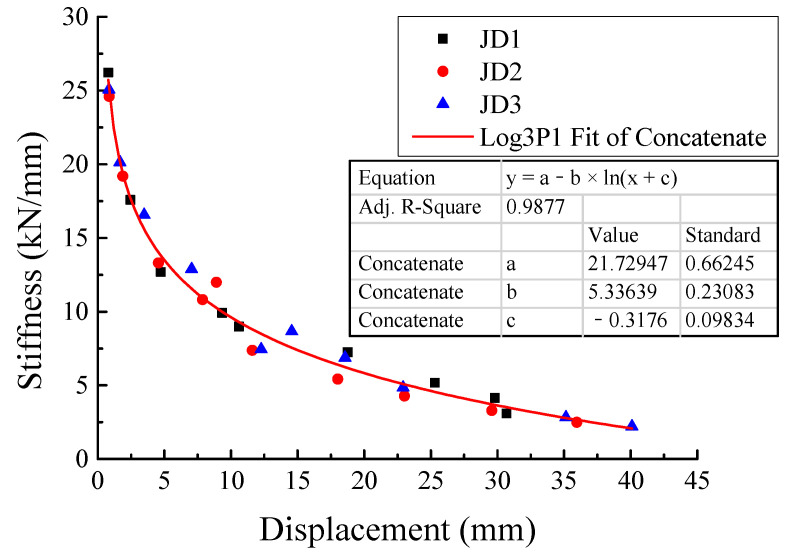
Stiffness degradation curves of specimens.

As shown in Equation (2), the secant stiffness of each step under low reversed cyclic loading was defined as the equivalent stiffness *K_i_*, where *F_i_* was the peak load of the step to be calculated, Δ*_i_* was the peak displacement of the step, and ‘+’ and ‘−’ represented the downward and upward directions, respectively. The equivalent stiffness values of every loading step are gathered in Table 5. Moreover, the stiffness distribution and variation trend are also illustrated in Figure 14. It can be seen from Table 5 and Figure 14 that the stiffness values of the PC specimens were very close to that of the CIP specimen. With the increasing displacement, all showed significantly similar degradation trends. By fitting these stiffness data of the three specimens, the evolution law of equivalent stiffness with increasing displacement can be obtained, as indicated in Figure 14.

(2) $$K_{i}=\frac{\left|+F_{i}\right|+\left|-F_{i}\right|}{\left|+\Delta_{i}\right|+\left|-\Delta_{i}\right|}$$

### 3.5. Energy Dissipation Performance

**Figure 15:**
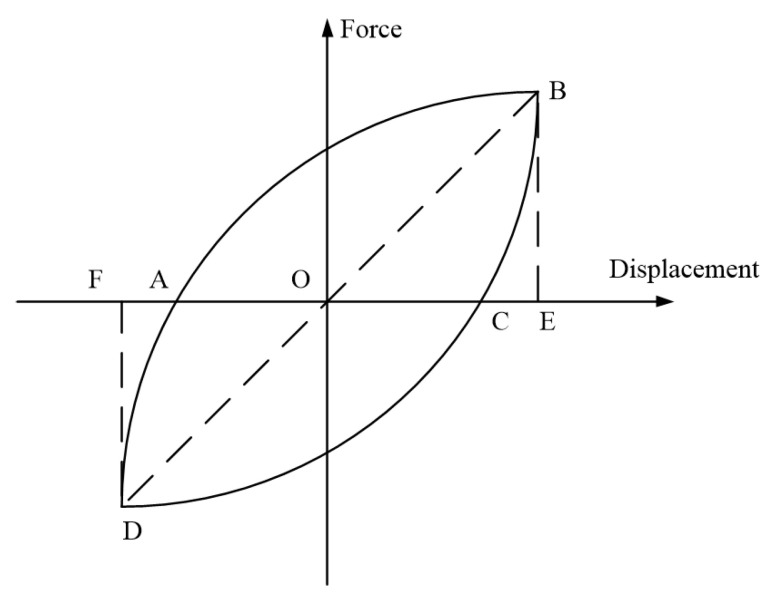
Calculation of equivalent viscous damping coefficient.

**Figure 16:**
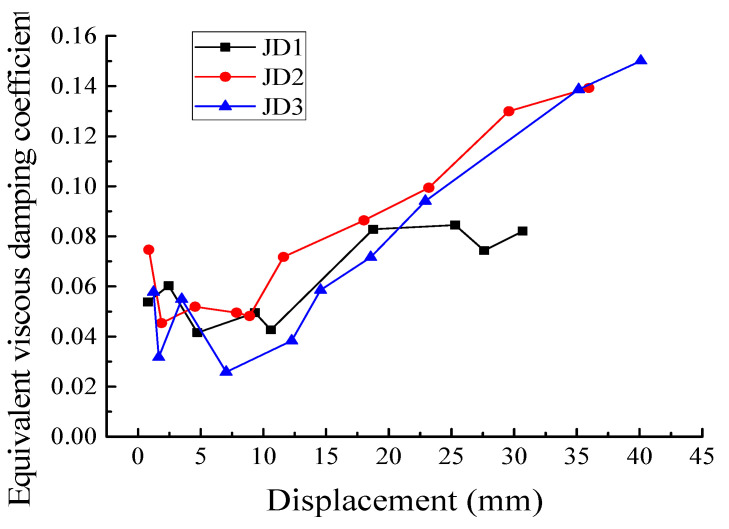
Evolution of equivalent viscous damping coefficient.

**Figure 17:**
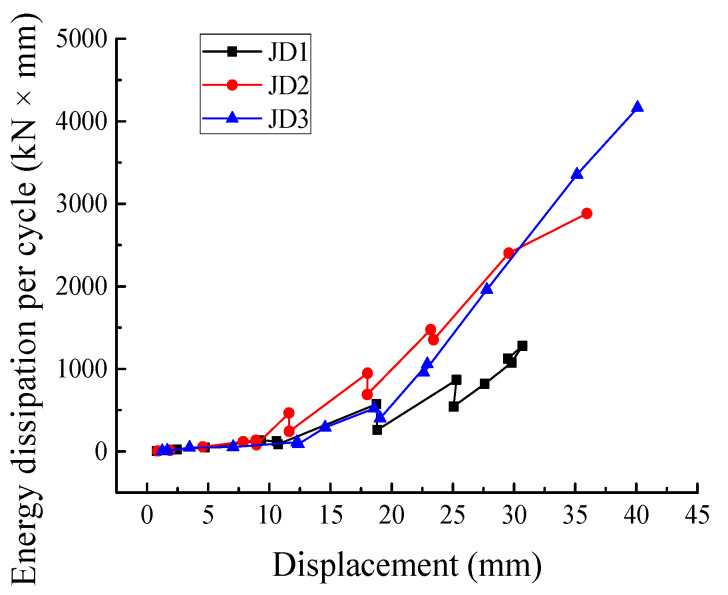
Energy dissipation per cycle for specimens.

**Figure 18:**
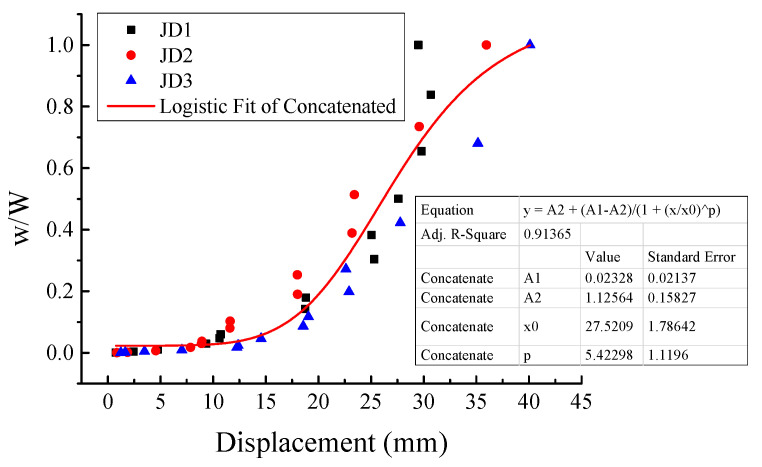
Evolution of normalized energy dissipation.

The energy dissipation properties of all the specimens under low reversed cyclic loading are shown in Figure 15, Figure 16, Figure 17 and Figure 18. According to Equation (3) [28], the equivalent viscous damping coefficient *ζ_eq_* is introduced to measure the energy dissipation capacity of the specimens.

(3) $$\zeta =\frac{1}{2\pi }\cdot \frac{S_{\left(ABC+CDA\right)}}{S_{\left(OBE+ODF\right)}}$$

As shown in Figure 15, S_(ABC+CDA)_ is the area enclosed by the hysteretic curve, and S_(OBE+ODF)_ is the sum area of triangle OBE and triangle ODF. It can be seen from Figure 16 that the equivalent viscous damping coefficients of the PC and CIP specimens showed similar evolution trends, all gradually increasing with the growth in the cycle number. The equivalent viscous damping coefficient of JD2 was greater than those of JD1 and JD3. Furthermore, after JD3 yielded, its equivalent viscous damping coefficient appeared slightly greater than that of JD1. These results indicated that the energy consumption capacity of the PC joints was slightly better than that of the CIP joint, and JD2 exhibited the best energy consumption capacity. Figure 17 presents the energy dissipation per cycle, which can be expressed as the area enclosed by each corresponding load–displacement hysteretic loop. The energy dissipation of the PC joints was not significantly different from that of the CIP joint before yielding, while it showed an obvious difference after yielding. In the middle and later phases of loading, the energy dissipation capacity of the specimens was improved; the improving speed of JD2 was obviously higher than that of JD1 and the energy dissipation of both JD2 and JD3 was larger than that of JD1. In general, the energy dissipation capacity of the PC specimens was better than that of the CIP specimen.

In order to study the evolution trend of the energy dissipation of the specimens, the normalized energy dissipation *w/W* was introduced for analysis [29]. Where *w* was defined as the cumulative hysteretic energy of the current cycle and previous cycles, i.e., *w = A_Cycle_*_1_ *+ A_Cycle_*_2_ *+* … *+ A_Cycle n_, W* was defined as the cumulative hysteretic energy of all cycles, i.e., *W = A_Cycle_*_1_ *+ A_Cycle_*_2_ *+* … *+ A_Cycle N_*, *A* denoted the area enclosed by each load–displacement hysteretic loop, and *n* and *N* denoted the number of the current cycle and the total cycles, respectively. The normalized energy dissipation per cycle and its evolution trend obtained by fitting the normalized energy dissipation data of all specimens are demonstrated in Figure 18. It can be seen that the energy dissipation evolution trend of the monolithic CIP specimen was close to that of the PC specimens. Before the specimens yielded, the energy dissipation was small and remained stable. After the specimens yielded, the energy dissipation rate increased rapidly. The energy dissipated in this phase accounted for the majority of the total energy. After entering the peak phase, the energy dissipation rate gradually flattened.

### 3.6. Strain of Longitudinal Steel Bars in Beam

According to the “Code for Design of Concrete Structures” [26], the length of the longitudinal steel bars in the beam of the CIP specimen anchored into the web of the T-shaped shear wall is calculated as shown in Equation (4).

(4) $$l_{ab}=\alpha \frac{f_{y}}{f_{t}}d$$

where *α* is the shape coefficient of the steel bars, which is taken as 0.14 for ribbed bars; *f_y_* is the design value of the steel bars’ tensile strength, which is taken as 360 N/mm^2^ for *HRB*400; *f_t_* is the design value of the concrete axial compressive strength, which is taken as 1.43 N/mm^2^ for *C*30; and *d* is the diameter of the steel bars. According to Equation (4), the anchorage length is 493 mm. In this test, it was designed as 570 mm. As for the PC specimens, the anchorage length of the longitudinal steel bars in the beams was consistent with that of the CIP specimen.

**Figure 19:**
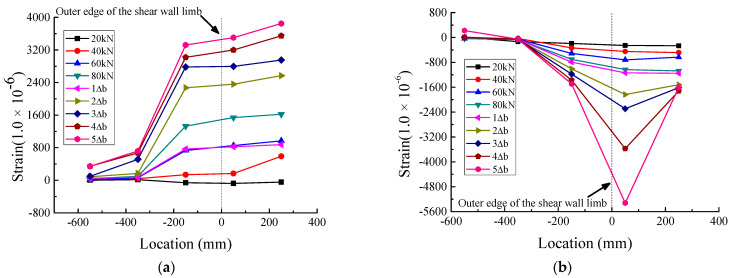
Strain distribution of longitudinal steel bars at the bottom of beam of JD1. (**a**) Tensile strain. (**b**) Compressive strain.

**Figure 20:**
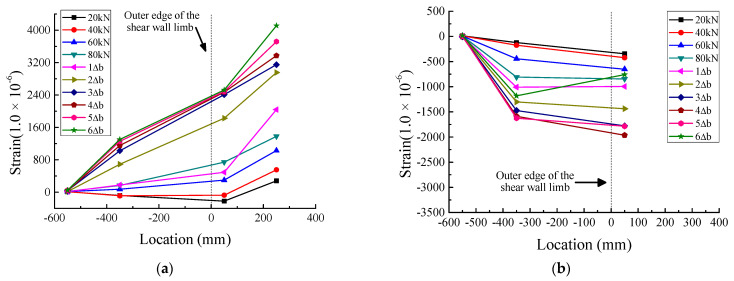
Strain distribution of longitudinal steel bars at the bottom of beam of JD2. (**a**) Tensile strain. (**b**) Compressive strain.

**Figure 21:**
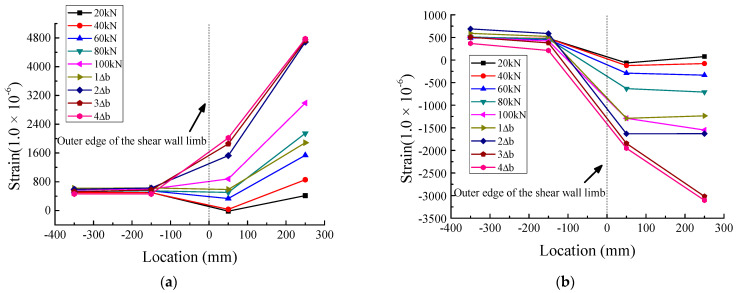
Strain distribution of longitudinal steel bars at the bottom of beam of JD3. (**a**) Tensile strain. (**b**) Compressive strain.

Figure 19, Figure 20 and Figure 21 show the strain distribution of the longitudinal steel bars at the bottom of the beam during loading. Since some strain gauges were damaged and invalidated gradually during the test, the values measured by these strain gauges are not plotted in the figures. The abscissa indicates the location of the strain gauges; the values express the distances between the strain gauges and the outer edge of the web of the T-shaped shear wall, which is defined as the zero point of the abscissa. Negative values indicate that the strain gauges were located in the anchorage section of the longitudinal steel bars, while positive values indicate that the strain gauges were located in the region where the longitudinal steel bars did not anchor into the web of the T-shaped shear wall. The ordinate represents the microstrain values.

Regarding JD1, as depicted in Figure 19, the tensile strains were very small at the location of −350 mm and varied slightly with the increase in load, and the tensile strains at −550 mm were slightly smaller than those at −350 mm. Furthermore, the compressive strains were extremely close to 0 at both −350 mm and −550 mm. This meant that the anchorage length of the longitudinal steel bars calculated according to Equation (4) was sufficient. As for JD2, it can be seen from Figure 20 that the tensile strains at −550 mm were much smaller than those at −350 mm*,* almost close to 0. Similar to the tensile strains, the compressive strains at −550 mm were also close to 0 and remained stable throughout the loading process. As shown in Figure 21, both the tensile strains and the compressive strains of JD3 were small and stable at −150 mm and −350 mm, respectively; all the strains were less than 800 με, which was lower than the corresponding strains of JD2. It can be seen that with the gradual increase in anchorage distance, the strains of the longitudinal steel bars of JD3 decreased faster than those of JD2. Besides, with the increase in anchorage distance, the PC specimens and the CIP specimen showed similar strain evolution trends; that is, the strain of the steel bars decreased continuously and gradually approached 0. This indicated that the anchorage length of the steel bars calculated by Equation (4) can meet the requirements of PC structures. 

## 4. Conclusions

In this study, three full-scale wall–beam–slab joint specimens were designed, and a quasistatic test was carried out by applying low reversed cyclic loading to the specimens. By comparing seismic performance indicators such as the failure mode, load-carrying capacity, ductility, and hysteretic loops of the PC joints with those of the CIP joint, the following conclusions can be drawn:

1. The overlapping U-bar loop connection adopted in assembling precast components is an effective and reasonable method. The stiffness of the PC joints was similar to that of the CIP joint; the load-carrying capacity, ductility, and energy dissipation of the PC joints were better than those of the CIP joint. Moreover, further experimental validations are still required to ensure that the mechanical properties of precast shear walls with this connection form can meet the seismic requirements.
2. By comparing the relevant mechanical indicators of JD2 with pure overlapping U-bar loop connections with those of JD3 with a modified form-overlapping U-bar loop connection combined with an extruded sleeve connection, it can be found that JD3 shows slightly better mechanical properties in general.
3. The failure mode of the PC joints was consistent with that of the CIP joint, which was marked by the buckling of the tensile steel bars, the crushing of concrete at the bottom of the beam, and a sharp decrease in load-carrying capacity. The generation, distribution, and development of cracks in the PC specimens were also similar to those in the CIP specimen. However, the precast-to-post-cast concrete interface between the web of the T-shaped shear wall and the beam cracked significantly during the loading process. Furthermore, there were fewer diagonal cracks distributed in the web of the T-shaped shear wall of the PC joints than that of the CIP joint.
4. The length of the longitudinal steel bars of the precast beam anchored into the web of the T-shaped shear wall was calculated in the same way as that of the CIP joint, so that it could meet the structural requirements of the PC joints. In the case where the requirements of anchor length were met, anchor damage did not occur.

## Figures and Tables

**Table 1:** Fabrication and connection methods of specimens.

Specimens	Type	Connection Methods Applied to Different Precast Components			
		Wall–Wall	Wall–Slab	Wall–Beam	Beam–Slab
JD1	CIP	integral	integral	integral	integral
JD2	PC	overlapping U-bar loop connections	overlapping U-bar loop connections	directly anchored	overlapping U-bar loop connections
JD3	PC	extruded sleeve connections (in confined boundary zone) overlapping U-bar loop connections (in intermediate region)	overlapping U-bar loop connections	directly anchored	overlapping U-bar loop connections

**Table 2:** Properties of concrete.

Type	Class	*f_cu_*/MPa
CIP specimen and precast components of PC specimens	C30	31.01
post-pouring concrete of PC specimens	C40	35.11

**Table 3:** Properties of steel bars.

Type	Diameter *D_s_* (mm)	Yield Strength *f_y_* (MPa)	Ultimate Strength *f_u_* (MPa)	Elastic Modulus *E_s_* (N/mm^2^)	Yield Strain *ε_y_* (10^−6^)
HRB400	10	563	660	2.028×10^5^	2776
HRB400	14	442	656	1.884×10^5^	2346

**Table 4:** Load-carrying capacity, displacement, and ductility values of specimens.

Specimen	Cracking Cycle		Yield Cycle		Peak Cycle		Ultimate Cycle		Ductility
	*F_cr_* (kN)	Δ*_cr_* (mm)	*F_y_* (kN)	Δ*_y_* (mm)	*F_p_* (kN)	Δ*_p_* (mm)	*F_u_* (kN)	Δ*_u_* (mm)	
JD1	39.505	2.45	79.468	9.34	111.786	29.8	93.567	30.68	3.285
JD2	39.490	1.88	79.437	7.86	124.252	23.20	98.175	35.96	4.575
JD3	39.612	1.26	79.346	7.04	121.023	35.14	106.522	40.10	5.696

**Table 5:** Secant stiffness values of specimens.

Specimen	Secant Stiffness										
	20 kN	40 kN	60 kN	80 kN	100 kN	1Δ_a_	2Δ*_a_*	3Δ*_a_*	4Δ*_a_*	5Δ*_a_*	6Δ*_a_*
JD1	26.219	17.581	12.696	9.912	-	8.99	7.245	5.171	4.141	3.095	-
JD2	24.602	19.196	13.311	10.812	-	11.997	7.382	5.424	4.275	3.289	2.495
JD3	25.063	20.139	16.566	12.885	7.445	8.666	6.875	4.847	2.826	2.203	-

## Data Availability

The data used to support the findings of this study are included within the article.
